# TEMPOROMANDIBULAR DYSFUNCTION, STRESS AND COMMON MENTAL DISORDER IN UNIVERSITY STUDENTS

**DOI:** 10.1590/1413-785220162406162873

**Published:** 2016

**Authors:** Viviane Gontijo Augusto, Keity Cristina Bueno Perina, Daniel Silva Gontijo Penha, Daiane Carolina Alves dos Santos, Valéria Aparecida Souza Oliveira

**Affiliations:** 1. Universidade do Estado de Minas Gerais, Unidade Divinópolis, Divinópolis, MG, Brazil.

**Keywords:** Temporomandibular joint. Stress, psychological. Mental disorders

## Abstract

**Objective:**

To evaluate the prevalence of temporomandibular dysfunction (TMD) and its association with perceived stress and common mental disorder (CMD) in academic students.

**Methods:**

This is s transversal observational study conducted at *Universidade de Minas Gerais*, Divinópolis Unit, in health science courses. To investigate the prevalence of TMD, the anamnestic index by Fonseca was used. Stress was assessed by the perceived stress scale, translated and adapted for the Brazilian population in 2006. To track CMD, we used the Self-Reporting Questionnaire (SRQ-20). Data were analyzed using SPSS version 13.0, adopting a 5% significance level.

**Results:**

The prevalence of TMD in the sample was 71.9%, distributed as follows: Light TMD (50.0%), moderate (16.4%) and severe (5.5%), being more frequent among women (76.4%). Common mental disorders were present in 29.9% of participants. The average perceived stress was 30.9.

**Conclusion:**

The results of this study allow us to conclude that there is a statistically significant correlation between TMD and variables such as parafunctional habits, perceived stress and CMD. Level of Evidence II, Development of diagnostic criteria on consecutive patients (with universally applied reference "gold" standard).

## INTRODUCTION

The temporomandibular joint (TMJ) is one of the most complex joints in the human body and it is responsible for opening and closing movements of the mouth and also the protrusion, retraction and lateral deviation movements of the mandible on the temporal bone. It is an extremely important structure, because its function is directly related to a context that involves communication, emotional expression and feeding, i.e., a set of factors that affect the individual's quality of life.^1^

Any imbalance caused in TMJ, or close to it structures, can generate a disturbance in this joint called Temporomandibular Disorder (TMD). The term is used to name clinical conditions of the joint, the masticatory muscles, and surrounding structures characterized by pain, joint sounds, and irregular function of the jaw. Pain is the main complaint of individuals with TMD, who may also experience symptoms such as muscle fatigue, tension headache and limited mandibular opening.^2^ Other common symptoms are sensitivity changes in the​ muscles responsible for chewing, noises during jaw movements, limitation or incoordination of movements and malposition of the jaw relative to the maxila.^3^

Among the risk factors for TMD there are the deleterious habits that escape from normal and harm an organ or system. Among them are: chewing gum, biting pencils, nail biting, gnashing or clenching. These habits can represent a way to release emotional tension. Parafunctions decrease normal blood flow of muscle tissue, causing accumulation of metabolic waste products in the cells of these tissues, triggering fatigue, pain and spasm symptoms.^4^ In general, parafunctions cause morphological and functional impairment of bones, teeth and soft tissue of stomatognathic system.

TMD may have other etiological factors such as poor dental occlusion and stress. There is evidence that stress leads to increased muscle activity causing pain to the region close to the temporomandibular joint.^5^ ^,^ ^6^

Chronic pain, such as those associated with TMD can produce not only biological effects but also psychological and social effects. Oliveira et al.^7^ assessed the impact of TMD in 22 patients assessed by physical therapists and concluded that more than half had work activities and study hampered by pain. It is also known that everyday activities, including social events, are potential sources of stress, because demand adaptation from the individual. This adaptation involves from changes in physiological processes in order to achieve behaviors adaptation. Thus, excess muscular activity may be associated with psychosocial aspects.^7^ ^,^ ^8^

Psychosocial aspects involve non-psychotic symptoms, such as insomnia, fatigue, irritability and somatic complaints are rarely diagnosed and comprise a group of disorders called Common Mental Disorders (CMD).

In general, the changes caused by TMD can interfere with daily life activities and social life of the individual, leading to a negative effect on his/her emotional and physical health, even in their academic and professional performance.^8^ The relationship between TMD, perceived stress and CMD is not yet clear and investigating the prevalence of these symptoms in university students may contribute to the development of preventive and rehabilitation strategies for this dysfunction.

Considering the facts exposed, the objective of this study was to evaluate the prevalence of temporomandibular dysfunction and its association with perceived stress and common mental disorders in college students.

## MATERIALS AND METHODS

This is a cross-sectional observational study conducted in health science courses of a higher education institution in Divinópolis, MG, Brazil. The sample was selected among volunteers in undergraduate students of health science courses in this institution who agreed to participate in the study after approval by the Ethics Research Committee of FUNEDI/UEMG under number 45878815.5.0000.5115. It included all students enrolled in one of the following courses: nursing, physiotherapy, psychology and physical education, who signed the Free and Informed Consent Term.

Students enrolled in health courses totaled 1,073 individuals. In order to determine the sample size, we used the procedures proposed by Luiz and Magnanini^9^ for finite populations. In this calculation, a 5% level of significance was adopted (corresponding to a confidence interval of 95%, z[α]/2 = 1.96) and tolerable error of the sample 5%, resulting in a required sample of 387 subject to estimate prevalence.

To evaluate the university students a protocol was used containing demographic data and specific instruments for evaluating TMD and perceived stress and mental disorder screening.

To investigate the prevalence of TMD, the Fonseca's anamnesis index was used.^10^ This index is a functional assessment scale, which contains 10 objective questions to assess whether the individual has temporomandibular joint dysfunction. Regarded as one of the few instruments that characterize the severity of symptoms of TMD, it allows the classification of the individual according to a score. No TMD (0-19), light TMD (20-44), moderate TMD (45-69) and severe TMD (70-100).^11^ The instrument has a good psychometric property.^11^

Stress was measured by the perceived stress scale, translated and adapted for the Brazilian population in 2006.^12^ The scale consists of 14 items and it measures the degree to which individuals perceive stress in daily situations. Response options range from zero to four. The scale presents questions with positive and negative emphasis related to stress and the total score ranges from 0 to 56. The higher the score, the greater the perceived stress.^12^

To trace the CMD we used the Self-Reporting Questionnaire (SRQ-20). This is an instrument recommended by the World Health Organization, particularly in developing countries, translated into eight different languages, which is easy to use and understand. The SRQ-20 version adapted for Brazil contains 20 questions and its score ranges from 0 to 20. Here as well, the higher the score, the greater the possibility of having the disorder. The cutoff point used was seven positive answers.^13^

Data were analyzed using SPSS version 13.0, adopting a 5% significance level. Descriptive analysis was made and the Pearson correlation test and chi-square test were used to verify associations between TMD, perceived stress and CMD.

## RESULTS

The sample represented 55% of the target population with the participation of 586 students, 450 (76.8%) female, with a mean age of 24±7 years old. Regarding marital status, 515 students (87.9%) were single. (Table 1) As for the evaluated parafunctions, the most common were leaning the chin over the hands, with 341 students reporting this habit (58.2%), followed by chewing gum, with 194 students (33.1%). (Table 2)

**Table 1:** Sociodemographic data of university students. Divinópolis, MG, Brazil, 2015. (n=586).

Variables	Frequency (n)	Percentage (%)
Gender		
Female	450	76.8%
Male	136	23.2%
Marital status		
Single	515	87.9%
Marries	62	10.6%
Divorced	8	1.3%
Widower	1	0.2%
Course		
Nursing	76	13%
Physical Education	102	17.4%
Physical Therapy	149	25.4%
Psychology	259	44.2%

**Table 2:** Results regarding parafunctional habits of university students. Divinópolis, MG, Brazil, 2015 (n = 586).

Variables	Affirmative answers	
	Frequency	Percentage
Biting nails	156	26.6%
Biting objects (pens, pencils)	171	29.2%
Lean the chin over the hand	341	58.2%
Chewing gum	194	33.1%
Bruxism	154	26.3%

 The prevalence of TMD in the sample was 71.9%, distributed as follows: light TMD (50.0%), moderate TMD (16.4%) and severe TMD (5.5%), being more frequent among women (76.4%) than men (57.4%). Common mental disorder was present in 29.9% of participants. In females, CMD was present in 33.8% of the sample, and only 18.4% of males.

The average perceived stress score was 30.9±6.0 and the median was 32. We used the median score to classify stress into high perceived stress (above the median) and low perceived stress (below the median), yielding almost half of the sample, 288 students (49.3%) with high perceived stress. The prevalence of CMD varied according to the severity of TMD, being more frequent among students with severe TMD. Similarly, the frequency of perceived stress was high in those with TMD. (Table 3)

**Table 3:** Correlation between the degree of TMD, perceived stress and CMD in university students, Divinópolis, MG, Brazil, 2015 (n = 586).

Variables	Intensity	no TMD	Mild TMD	Moderate TMD	Severe TMD
Stress	Low	111(67.7%)	174(59.2%)	60(61.5%)	16(50%)
High	53 (32.3%)	119(40.8%)	37(38.5%)	16(50%)	
CMD	Absent	147(89.6%)	201(68.8%)	49(51%)	12(37.5%)
Present	17(10.4%)	92(31.2%)	47(49%)	21(62.5%)	

Of the 586 participants, 245 (42.0%) did not use braces. Of the remaining 341, 104 (17.8%) have used them and 235 (40.2%) was still using. Therefore, 58.0% of the sample have used or was using orthodontic appliances. The average usage time was 16.8±24.3 months. 

The Pearson correlation test showed a weak although significant correlation between TMD and perceived stress (r=0.11, *p*=0.005). It was also possible to observe a moderate correlation between TMD and common mental disorder (r=0.46, *p*=0.000). The chi-square test showed a significant association between TMD and parafunctional habits like biting pens (*p*=0.002), leaning the chin over the hands (*p*=0.018) and bruxism (*p*=0.000).

## DISCUSSION

The results of this study reinforce the assumption that there is an association between TMD and parafunctional habits such as perceived stress and CMD. Although the association between psychological factors and TMD is inconsistent in the literature, there is biological plausibility for this association.^14^ According to Kindler et al.,^15^ psychological factors can initiate muscle hyperactivity, followed by biomechanical changes and consequent pain. They can also produce neurotransmitters serotonin and catecholamines imbalance, inducing pain. Moreover, pain in the temporomandibular region can be the physical manifestation of CMD.^16^ TMD patients are anxious, perfectionist, dominators and tend to express their anxiety through physical symptoms. In these individuals, apprehension, frustration, hostility and fear are common feelings.^16^

The high prevalence of signs and symptoms of TMD (71.9%) confirms the results by Oliveira et al.^17^ conducted with college students in Brazil, where the prevalence of TMD was 68.6%. However, there are studies showing that TMD was less common among college students, as the study by Minghelli et al.,^18^ who evaluated university students in the health area, finding TMD in only 37.3%. These differences may be related to the variety of courses studied, the life context, and the academic status of the students at the time of the survey.

Regarding parafunctional habits the most prevalent were leaning the chin over the hands and chewing gum, corroborating the study of Medeiros et al.,^19^ who also found a high prevalence of these habits. It is believed that parafunctional habits are an unconscious means to release tension and this may being consciously or unconsciously, happening during sleep and wakefulness, contributing to the onset of temporomandibular disorders or its perpetuation.^20^

It is important to mention that we observed a higher prevalence of TMD among women. This fact has been attributed to the presence of female hormones that interfere with pain threshold.^19^ According to Menezes,^20^ women's levels of estrogen may explain the higher tissue flaccidity of joints, leading to lower ability to withstand functional pressure.

This study found that almost 60% of the sample have used or was using braces. Some studies attempted to explain the possible relationship between orthodontics and temporomandibular disorders. Despite significant advances, this relationship could not be fully understood and there is still controversy among researchers. While some claim that orthodontic correction can heal TMD, others sustain that orthodontic devices can cause pain and dysfunction of the stomatognathic system.^21^ ^,^ ^22^

The presence of CMD in one third of the sample is also noteworthy, since these disorders affect several areas of functioning, including academic performance. This disorder is also more common among women, which reinforces the assumption that female are more prone to develop non-psychotic psychological symptoms.^23^

The participants in this study had a high score of perceived stress (30.9±6.0). This result is close to the values ​​found by Faro,^24^ who evaluated 2,157 *lato sensu* (masters and doctoral) graduate students in Brazil through the same scale of perceived stress, and found a mean score of 29.1 points.

It is possible that the level of perceived stress in this sample was high due to the constant assessment, preparation of assignments and reports, that are part of academic life. Interpersonal and intrapersonal relationships may have also been reason for stress, besides limited time to accomplish tasks related to family, work and school, possible financial problems increasingly constant in the world nowadays. Of course, one should consider that students from every educational institution experience, at various levels of intensity, stress during the learning process and depend on the reality they live in, because fluctuations of the stress intensity may occur during education years.

Finally, we observed a trend towards more psychosocial factors (perceived stress and CMD) in severe TMD cases, and this may indicate that such factors are aggravating TMD. However, because this is a cross-sectional study, it is difficult to establish a temporal relationship between events and it is not possible to assert a causal relationship between them.

## CONCLUSION

The results of this study allow us to conclude that parafunctional habits, perceived stress and CMD correlate with TMD, although it is not possible to establish a causal relationship between the events. Therefore, it is not possible to state that the presence of deleterious habits, high perceived stress and CMD signals are causes of TMD among the participants, but the existing correlation between these factors paths the way for preventive actions aimed at students, especially those with moderate and severe signs of TMD.

An interdisciplinary extension project could be created to this audience, which may include activities such as conversation circles and practices related to oral care, reducing parafunctional habits, perceived stress and CMD.
